# Supplementary material for: β-Arrestin-independent endosomal cAMP signaling by a polypeptide hormone GPCR
Source: Nat Chem Biol. 2023 Sep 25;20(3):323–32. doi: 10.1038/s41589-023-01412-4 (PMC10907292; doi:10.1038/s41589-023-01412-4)
Supplement: Supplementary file 1 — Supplementary Fig. 1 and references. [file 41589_2023_1412_MOESM1_ESM.pdf]

nature chemical biology

Article

https://doi.org/10.1038/s41589-023-01412-4

β-Arrestin-independent endosomal cAMP signaling by a polypeptide hormone GPCR

In the format provided by the authors and unedited

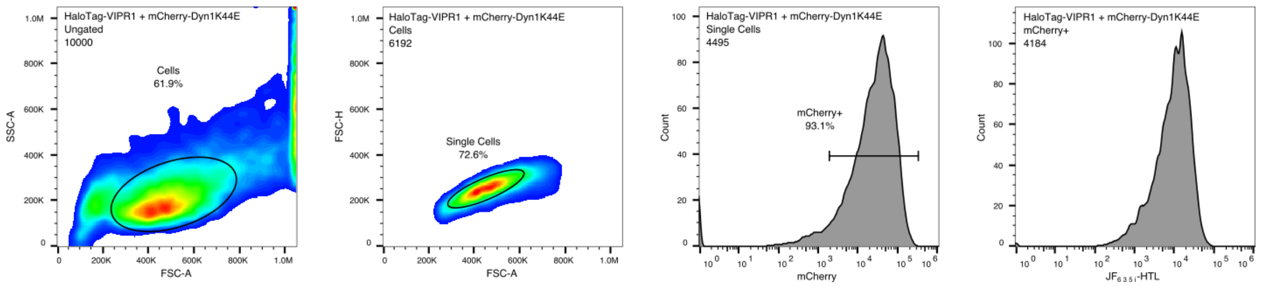

Supplementary Fig. 1 Example flow cytometry gating strategy. Gating strategy for cells co-expressing HaloTag-VIPR1 and mCherry/mCherry-Dyn1K44E is shown. For all other experiments with cells expressing only a HaloTag receptor, the gating strategy is the same, excluding the mCherry+ gate.

References

1. Peng, G. E., Pessino, V., Huang, B. & von Zastrow, M. Spatial decoding of endosomal cAMP signals by a metastable cytoplasmic PKA network. Nat. Chem. Biol. 17, 558–566 (2021).
2. Xu, Z. et al. Structural basis of sphingosine-1-phosphate receptor 1 activation and biased agonism. Nat. Chem. Biol. 18, 281–288 (2022).
3. Barsi-Rhyne, B., Manglik, A. & von Zastrow, M. Discrete GPCR-triggered endocytic modes enable β-arrestins to flexibly regulate cell signaling. eLife 11, e81563 (2022).
4. Wan, Q. et al. Mini G protein probes for active G protein–coupled receptors (GPCRs) in live cells. J. Biol. Chem. 293, 7466–7473 (2018).
5. Dixon, A. S. et al. NanoLuc Complementation Reporter Optimized for Accurate Measurement of Protein Interactions in Cells. ACS Chem. Biol. 11, 400–408 (2016).
6. Cao, T. T., Deacon, H. W., Reczek, D. & Bretscher, A. A kinase-regulated PDZ-domain interaction controls endocytic sorting of the b2-adrenergic receptor. 401, 5 (1999).
7. Eichel, K., Jullié, D. & von Zastrow, M. β-Arrestin drives MAP kinase signalling from clathrin-coated structures after GPCR dissociation. Nat. Cell Biol. 18, 303–310 (2016).
