# Supplementary material for: β-Arrestin-independent endosomal cAMP signaling by a polypeptide hormone GPCR
Source: Nat Chem Biol. 2023 Sep 25;20(3):323–32. doi: 10.1038/s41589-023-01412-4 (PMC10907292; doi:10.1038/s41589-023-01412-4)
Supplement: Supplementary file 2 — Reporting Summary [file 41589_2023_1412_MOESM2_ESM.pdf]

nature portfolio

Corresponding author(s): Mark von Zastrow

Last updated by author(s): 07/05/2023

Reporting Summary

Nature Portfolio wishes to improve the reproducibility of the work that we publish. This form provides structure for consistency and transparency in reporting. For further information on Nature Portfolio policies, see our Editorial Policies and the Editorial Policy Checklist.

Statistics

For all statistical analyses, confirm that the following items are present in the figure legend, table legend, main text, or Methods section.

n/a	Confirmed	
☐	☒	The exact sample size (*n*) for each experimental group/condition, given as a discrete number and unit of measurement
☐	☒	A statement on whether measurements were taken from distinct samples or whether the same sample was measured repeatedly
☐	☒	The statistical test(s) used AND whether they are one- or two-sided<br>*Only common tests should be described solely by name; describe more complex techniques in the Methods section.*
☐	☒	A description of all covariates tested
☐	☒	A description of any assumptions or corrections, such as tests of normality and adjustment for multiple comparisons
☐	☒	A full description of the statistical parameters including central tendency (e.g. means) or other basic estimates (e.g. regression coefficient) AND variation (e.g. standard deviation) or associated estimates of uncertainty (e.g. confidence intervals)
☐	☒	For null hypothesis testing, the test statistic (e.g. *F*, *t*, *r*) with confidence intervals, effect sizes, degrees of freedom and *P* value noted<br>*Give P values as exact values whenever suitable.*
☒	☐	For Bayesian analysis, information on the choice of priors and Markov chain Monte Carlo settings
☒	☐	For hierarchical and complex designs, identification of the appropriate level for tests and full reporting of outcomes
☐	☒	Estimates of effect sizes (e.g. Cohen's *d*, Pearson's *r*), indicating how they were calculated

Our web collection on statistics for biologists contains articles on many of the points above.

Software and code

Policy information about availability of computer code

Data collection	NIS Elements HC v5.21.03 (Nikon), Micromanager v1.4.23, Gen5 v2.05 (BioTek), Attune Cytometric Software v5.3.2415.0 (Thermo Fisher), Odyssey v2.0.3 (LI-COR Biosciences)
Data analysis	Cell Profiler 4, Fiji v1.53, FlowJo v10.8 (BD Life Sciences), Prism v8 and v9 (GraphPad), Synthego ICE Analysis Tool

For manuscripts utilizing custom algorithms or software that are central to the research but not yet described in published literature, software must be made available to editors and reviewers. We strongly encourage code deposition in a community repository (e.g. GitHub). See the Nature Portfolio guidelines for submitting code & software for further information.

Data

Policy information about availability of data

All manuscripts must include a data availability statement. This statement should provide the following information, where applicable:

- Accession codes, unique identifiers, or web links for publicly available datasets
- A description of any restrictions on data availability
- For clinical datasets or third party data, please ensure that the statement adheres to our policy

Data and uncropped blots for Figs. 1–5 and Extended Data Figs. 1–2,4-8 are provided with this paper as source data files.

Human research participants

Policy information about studies involving human research participants and Sex and Gender in Research.

Reporting on sex and gender	Not applicable.
Population characteristics	Not applicable.
Recruitment	Not applicable.
Ethics oversight	Not applicable.

Note that full information on the approval of the study protocol must also be provided in the manuscript.

Field-specific reporting

Please select the one below that is the best fit for your research. If you are not sure, read the appropriate sections before making your selection.

☒ Life sciences ☐ Behavioural & social sciences ☐ Ecological, evolutionary & environmental sciences

For a reference copy of the document with all sections, see nature.com/documents/nr-reporting-summary-flat.pdf

Life sciences study design

All studies must disclose on these points even when the disclosure is negative.

Sample size	Sample sizes were chosen according to the current standards of the field and verified by experience in our lab in the present experimental system (ex. PMID 33649598). For all experiments, at least three independent biological replicates, defined as samples from separate preparations on different days, were analyzed. Each biological replicate represents the mean of at least two technical replicates, defined as samples from the same preparation analyzed on the same day.
Data exclusions	No data were excluded from analysis.
Replication	All experiments were carried out at least three times, and all findings in the manuscript were reproducible.
Randomization	This is not relevant to the study because all experiments were done in the same cell line background.
Blinding	Blinding was not done during data collection or analysis. Blinding is not relevant as most of the data collection and analysis methods are not vulnerable to human bias. The only results potentially subject to human bias are the microscopy-based colocalization experiments. Accordingly, all colocalization experiments were verified using an orthogonal method (NanoBiT) that is inherently unbiased.

Reporting for specific materials, systems and methods

We require information from authors about some types of materials, experimental systems and methods used in many studies. Here, indicate whether each material, system or method listed is relevant to your study. If you are not sure if a list item applies to your research, read the appropriate section before selecting a response.

Materials & experimental systems

n/a	Involved in the study	
☐	☒	Antibodies
☐	☒	Eukaryotic cell lines
☒	☐	Palaeontology and archaeology
☒	☐	Animals and other organisms
☒	☐	Clinical data
☒	☐	Dual use research of concern

Methods

n/a	Involved in the study	
☒	☐	ChIP-seq
☐	☒	Flow cytometry
☒	☐	MRI-based neuroimaging

Antibodies

Antibodies used	anti-b-arrestin1/2 (Cell Signaling Technologies cat# 4674, clone D24H9)<br>anti-tubulin (Cell Signaling Technologies cat# 3873, clone DM1A)<br>IRDye 680RD Donkey anti-Mouse IgG (LI-COR Biosciences cat# 926-68072)

	IRDye 800CW Donkey anti-Rabbit IgG (LI-COR Biosciences cat# 926-32213)<br>anti-FLAG (Sigma Aldrich cat# F3040, clone M1)<br>anti-LgBiT (Promega cat# N7100)<br>anti-EEA1 (Santa Cruz Biotechnology cat# sc-6415)<br>Donkey anti goat IgG AlexaFluor 488 (Invitrogen cat# A-11055)<br>Donkey anti mouse IgG AlexaFluor 647 (Invitrogen cat# A-31571)
Validation	All antibodies are commercially available and have been previously validated/published, according to manufacturer's websites:<br>anti-b-arrestin1/2: https://www.cellsignal.com/products/primary-antibodies/b-arrestin-1-2-d24h9-rabbit-mab/4674<br>anti-tubulin: https://www.cellsignal.com/products/primary-antibodies/a-tubulin-dm1a-mouse-mab/3873<br>IRDye 680RD Donkey anti-Mouse IgG: https://www.licor.com/bio/reagents/irdye-680rd-donkey-anti-mouse-igg-secondary-antibody<br>IRDye 800CW Donkey anti-Rabbit IgG: https://www.licor.com/bio/reagents/irdye-800cw-donkey-anti-rabbit-igg-secondary-antibody<br>anti-FLAG: https://www.sigmaaldrich.com/US/en/product/sigma/f3040<br>anti-LgBiT: https://www.promega.com/products/protein-detection/primary-and-secondary-antibodies/anti-lgbit-monoclonal-antibody/?catNum=N7100<br>anti-EEA1: https://www.scbt.com/p/eea1-antibody-n-19<br>Donkey anti goat IgG AlexaFluor 488: https://www.thermofisher.com/antibody/product/Donkey-anti-Goat-IgG-H-L-Cross-Adsorbed-Secondary-Antibody-Polyclonal/A-11055<br>Donkey anti mouse IgG AlexaFluor 647: https://www.thermofisher.com/antibody/product/Donkey-anti-Mouse-IgG-H-L-Highly-Cross-Adsorbed-Secondary-Antibody-Polyclonal/A-31571

Eukaryotic cell lines

Policy information about cell lines and Sex and Gender in Research

Cell line source(s)	All cell lines, including VIPR1 and β-arrestin knockout cell lines, were derived from HEK293 cell line from ATCC (CRL-1573), except for GRK2/3/5/6 KO cell line which is from A. Inoue (Kawakami, K. et al. Heterotrimeric Gq proteins act as a switch for GRK5/6 selectivity underlying β-arrestin transducer bias. Nat. Commun. 13, 487 (2022)).
Authentication	Not authenticated.
Mycoplasma contamination	All cell lines tested negative for mycoplasma.
Commonly misidentified lines<br>(See ICLAC register)	No commonly misidentified lines were used.

Flow Cytometry

Plots

Confirm that:

☒ The axis labels state the marker and fluorochrome used (e.g. CD4-FITC).

☒ The axis scales are clearly visible. Include numbers along axes only for bottom left plot of group (a 'group' is an analysis of identical markers).

☒ All plots are contour plots with outliers or pseudocolor plots.

☒ A numerical value for number of cells or percentage (with statistics) is provided.

Methodology

Sample preparation	HEK293 cultured cells were enzymatically lifted. When appropriate, cells were labeled with HaloTag ligands.
Instrument	Attune NxT (Thermo Fisher)
Software	Attune NxT Software (Thermo Fisher)
Cell population abundance	HEK293 cultured cells stably overexpressing GPCRs of interest were used, and as such, no gating was needed to select for receptor-positive cells. When cells were also overexpressing mCherry or mCherry-Dyn1K44E via BacMam, cells were gated for mCherry expression.
Gating strategy	FSC-A vs. SSC-A and FSC-A vs. FSC-H gates were used to identify single cell populations. In the case where mCherry(Dyn1K44E) was also expressed, an mCherry+ gate was used to select for cells with similar levels of mCherry fluorescence.

☒ Tick this box to confirm that a figure exemplifying the gating strategy is provided in the Supplementary Information.
