# Supplementary figures and images for: β-Arrestin-independent endosomal cAMP signaling by a polypeptide hormone GPCR
Source: Nat Chem Biol. 2023 Sep 25;20(3):323–32. doi: 10.1038/s41589-023-01412-4 (PMC10907292; doi:10.1038/s41589-023-01412-4)

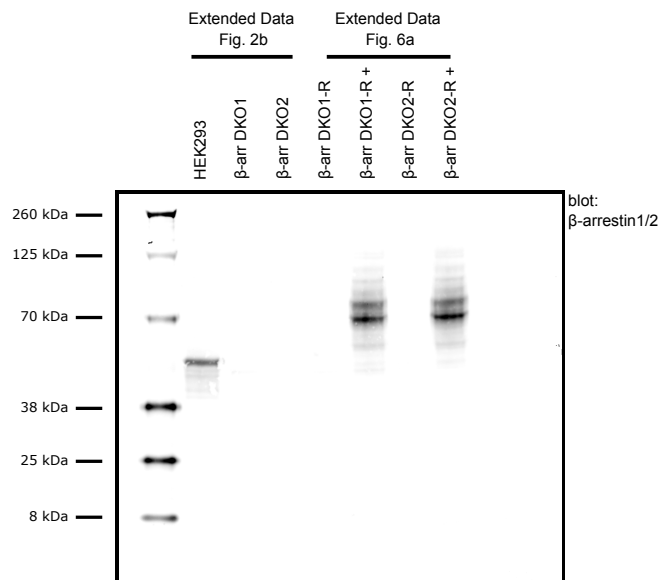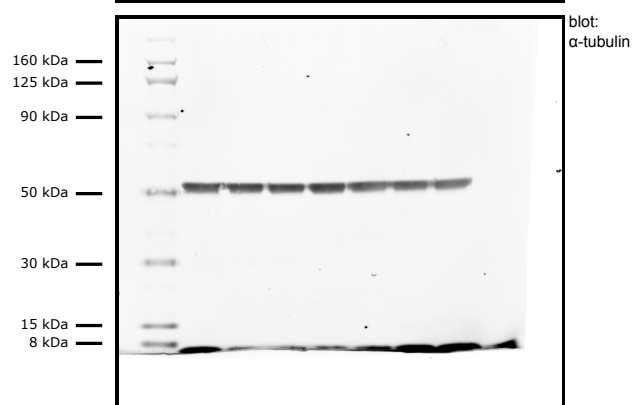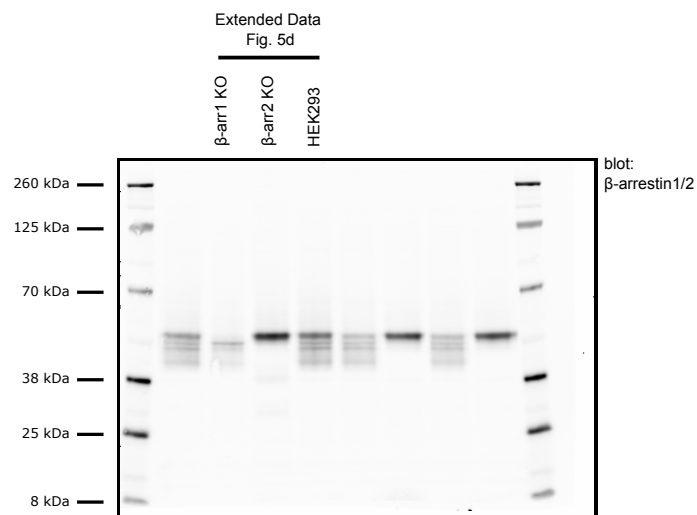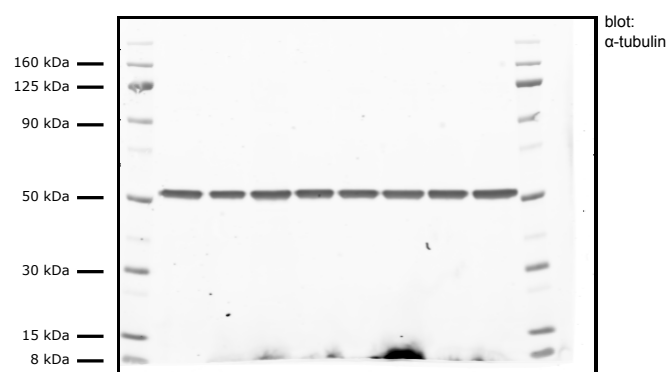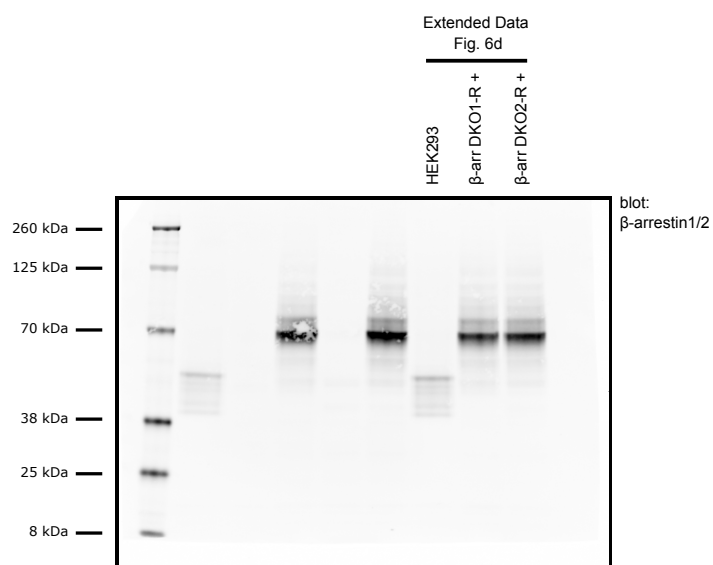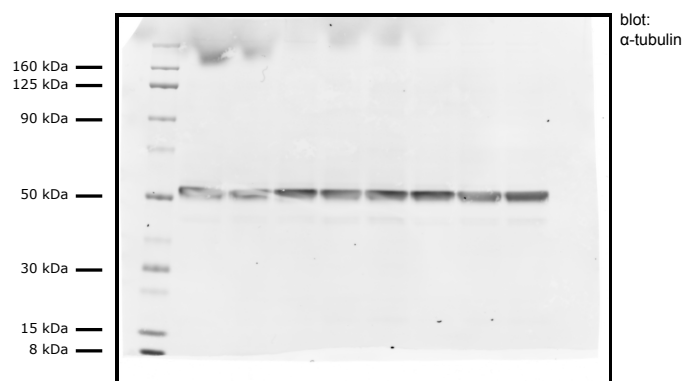

Supplement: Supplementary file 16 — Unprocessed western blots. [file 41589_2023_1412_MOESM16_ESM.pdf]
